# Effect of camera monitoring and feedback along with training on hospital infection rate in a neonatal intensive care unit

**DOI:** 10.1186/s12941-019-0332-y

**Published:** 2019-11-13

**Authors:** Meltem Karabay, Gulsum Kaya, Taner Hafizoglu, Oguz Karabay

**Affiliations:** 1 Department of Pediatrics, Newborn Unit, Sakarya University Faculty of Medicine, Sakarya, 54100 Türkiye; 2 Member of Infection Control Committee, Sakarya University Faculty of Medicine, Adnan Menderes Bulvari, Sakarya, Turkey

**Keywords:** Healthcare-associated infections, Neonatal, Training, Camera monitoring and feedback

## Abstract

**Background:**

In terms of pediatric healthcare-associated infections (HAI), neonatal intensive care units (NICU) constitute the greatest risk. Contacting a health care personnel, either directly or indirectly, elevates NICU occurrence rate and risks other infants in the same unit. In this study, it is aimed to retrospectively analyze the effect of the training along with camera monitoring and feedback (CMAF) to control the infection following a small outbreak.

**Methods:**

ESBL producing *Klebsiella pneumoniae* was detected on three infants in May 2014 at the isolation room of Sakarya University Hospital NICU. Precautions were taken to prevent further spread of the infection. The infected infants were isolated and the decolonization process was initiated. For this aspect, health care workers (HCWs) in NICU were trained for infection control measures. An infection control committee has monitored the HCWs. Before monitoring, an approval was obtained from the hospital management and HCWs were informed about the CMAF, who were then periodically updated. On a weekly basis, NICU workers were provided with the feedbacks. Epidemic period and post-epidemic control period (June–July–August 2014) were evaluated and p value < 0.05 was considered statistically significant.

**Results:**

Healthcare-associated infection (HAI) density was 9.59% before the onset of the CMAF, whereas it was detected as 2.24% during the CMAF period (p < 0.05). Following the precautions, HAI and HAI density rates have reduced to 76.6% and 74.85%, respectively. Moreover, hand hygiene compliance of health care workers was found 49.0% before the outbreak, whereas this rate has elevated to 62.7% after CMAF.

**Conclusions:**

Healthcare workers should be monitored in order to increase their compliance for infection control measures. Here, we emphasized that that CMAF of health workers may contribute reducing the HAI rate in the NICU.

## Background

Neonatal intensive care units (NICU) are the riskiest units in healthcare-associated infections (HAIs). HAIs in newborn infants constitute great danger in terms of morbidity, mortality, cost and long-term sequelae. Invasive procedures (central catheters, endotracheal tubes, etc.) are frequently applied to neonates in these units. Infants with low birth weights (< 1500 g), congenital abnormalities, umbilical or central catheter, long term stay in NICU, parenteral nutrition, broad spectrum antibiotic usage were potential causes of HAIs [1]. Since newborns has not developed sufficient immunity, HAIs may lead to life-threatening problems [2].

Studies demonstrated that pathogen microorganisms were initially detected on NICU surfaces, which is then followed by the guts of infants, with the most probable reservoir being tubing (e.g., nasogastric feeding tube), and their incubators and sinks [3, 4]. Direct or indirect contact with an infected or colonized health care workers (HCW) or the transition from one infant to another are crucial risks of NICU infections [5, 6]. Indirect infectious transmissions in NICUs might be caused by non-decontaminated devices, aspiration devices, rectal thermometers, umbilical cord solutions, superficial materials, which are mostly contaminated by hands. NICU outbreaks usually originate from multiple sources. For this reasons, single source cannot be responsible for the eradication of bacteria from NICU setting [7]. Transmission of responsible bacteria mainly occurs through direct contact with HCWs. Thus, in order to prevent nosocomial NICU infection, we should aim to overcome direct contact between infants and colonized individuals. Total parenteral nutrition fluid is the most common source of infection [3]. Intravenous fluids become more contaminant in the hands of HCWs while being used. Formulas might be contaminated during preparation. Infected HCWs might spread pathogenic microorganisms in NICUs [8]. These situations also increase the risk of HAIs in those infants [9]. To minimize outbreaks in the NICU, each newborn should be treated as if it contains unique flora colonies that should not be transferred to other neonates [10].

Several practices such as isolation of infected patient, expansion of compliance with hand hygiene, increased environmental cleanliness, and efficient catheter maintenance have been demonstrated to reduce HAIs in NICUs [11]. However, HAIs are still major problem among preterm infants in NICU. Additionally, HAIs are often seen as epidemic in the NICU. In outbreak conditions, NICU patients are particularly vulnerable to colonization and infection with pathogens such as multidrug resistant, Gram-negative bacteria [12]. Studies have reported that prematurity and long term antibiotic treatments were defined as independent risk factors during multidrug resistant Gram negative bacilli outbreak in NICU [12, 13]. Dynamic measures must be applied quickly in an outbreak [14, 15]. To overcome the outbreaks and control the eradication of the infectious bacteria in NICU, multidisciplinary interventions based on standard infection prevention practices are critically needed [7].

We recently had an outbreak experience in our newborn unit. During this outbreak, we have taken various measures to overcome the outbreak rapidly. One of the most important precautions was camera tracking. This study aims to evaluate the efficacy of the camera monitoring and feedback (CMAF) along with the infection control training after an epidemic in NICU.

## Methods

### Place

This retrospective cross-sectional study was conducted in Sakarya University Training and Research Hospital NICU with total of 17 bed capacity. The NICU staff included 2 doctors, 23 nurses, 4 fellows and 4 other HCWs. During the outbreak, NICU was in full capacity in terms of hospitalized infants. Nosocomial infections were detected on 3 infants when the outbreak occurred in May 2014.

### Before CMAF period

In this period, *K. pneumoniae* was isolated from blood cultures of 3 newborns in NICU.

### Outbreak period

During this period, health care workers at NICU were observed with camera monitoring and trained for infection control measurements.

Epidemic NICU was followed in the third level on May 2014. At the same time, unexpectedly, three new cases of general condition deterioration, circulatory failure, respiratory distress, abdominal distension were detected as well. *K. pneumoniae* was isolated from three reciprocal blood cultures. The infection control committee gave an outbreak alarm to prevent the spread of the bacteremia and the unit was taken into the quarantine.

### Post-epidemic period

Following the epidemic period, post-epidemic control period was introduced on June–July–August 2014. During this period, NICU HCWs continued to be monitored. Additionally, above-mentioned HCWs were trained on HAI and prevention strategies.

All NICU rooms were cleaned and the staff were educated on completing the terminal disinfection. Blood samples were collected from all possible infected infants. The insulation precautions were applied carefully on each patient. Antibiotic usage was requested in accordance with the recommendations of Hospital Infection Control Committee. Proceeding the approval from the hospital administration, the staff were informed and a 24-h recording has been obtained by a camera. Babies were immediately isolated in order to prevent contaminating other infants.

### Camera monitoring (CM)

Official consent was obtained from the hospital administration during the epidemic. Prior to the initiation of camera monitoring, all HCWs were clearly informed about the recording. After the outbreak, sanitation precautions were reinforced. The infection control committee, hospital management and neonatal department collaborated for the epidemic. Audits and observations were increased in the NICU and camera tracking was performed for 24 h. The obtained video recordings were then evaluated by the infection control committee. Both positive and negative behaviors captured by CMAF in the NICU were extensively discussed with the personnel. During this session, the employees were de-identified and anonymized. Thus, all actions were examined in confidentiality.

### Feedback

We conducted the study with sensitivity in order not to affect any staff psychologically. For this purpose, no staff was informed about their colleagues’ fault. Before the feedback, each HCW was anonymized on the camera recordings, in which the staff became unidentified. All errors that the personnel made were monitored. On the last day of each week, all good and bad actions detected by the camera were reported to the people, who were in charge of the units. The unit heads then in turn informed the HCWs.

### Training

In July 2014, 1 month after the outbreak, entire personnel was trained for infection prevention by infection control team of the hospital.

This theoretical training included transmission ways of nosocomial infections, importance of hand washing and hand hygiene, and ways of preventing the nosocomial infections. The training was provided by Oğuz Karabay and Gülsüm Kaya (members of infection control team). All of these trainings were accredited by provincial health directorate. The content of this training included hand hygiene practices, HAI factors and intensive care unit epidemics.

### Hand hygiene compatibility

Hand hygiene compliance had been measured in working hours since 2008. Three trained observers had monitored the measurements. An observation form was established according to five indications in hand hygiene, in which it was assessed that patients were first contacted before the aseptic procedure, after the risk of exposure to body fluids, after contacting the patient, after contacting the contaminated environment and after hand hygiene [16].

### Diagnosis of HAIs

The infection and colonization discrimination of the babies were performed according to the diagnostic criteria of Center for Diseases Control (CDC) HAIs. In the sub-distribution of HAIs, laboratory-induced bloodstream infection, central venous catheter-related bloodstream infections, urinary catheter-related urinary tract infections and ventilator-related bloodstream infections were established according to the diagnostic criteria of CDC.

### HAI rate measurement

The rate of HAI was calculated by dividing the number of HAIs developed in the clinic by the number of hospitalized patients at that time.

### HAI density

The number of HAIs that developed during this period was divided by the total inpatient days and multiplied by 1000.

### Ethical approval

The ethical approval of this study was obtained from Sakarya University Faculty of Medicine Ethics Committee (21.01.2015/17).

### Statistics

Descriptive statistics were used to compare the initial and subsequent characteristics of the study periods. Two variant analysis were performed to assess the differences between pre- and post-intervention. The Chi square test was used for binary variables. A p value < 0.05 was considered statistically significant. All data were analyzed with Epi-Info program (CDC, Atlanta, USA).

## Results

**Fig. 1:**
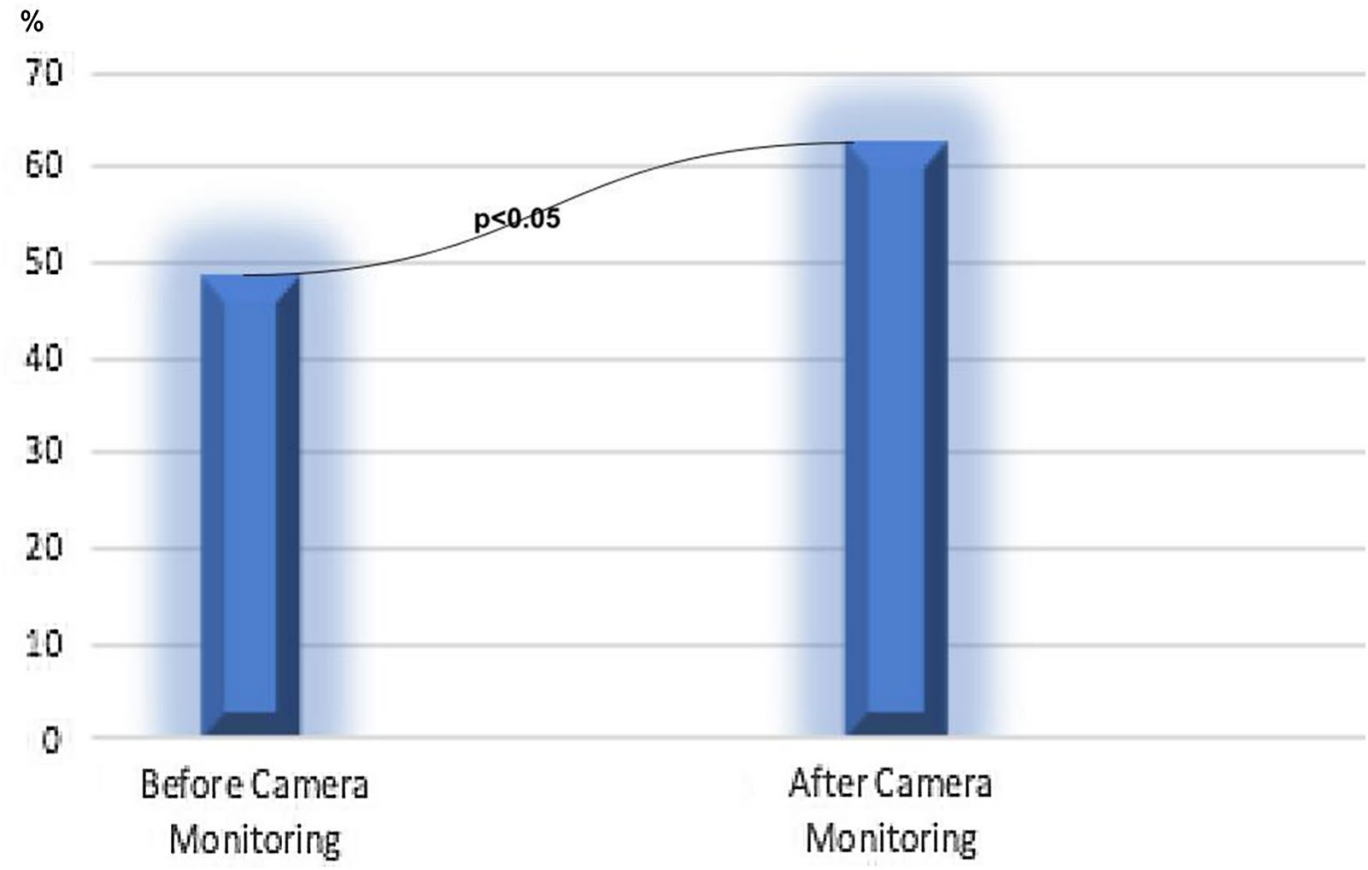
Hand hygiene compliance before and after the camera observation

**Fig. 2:**
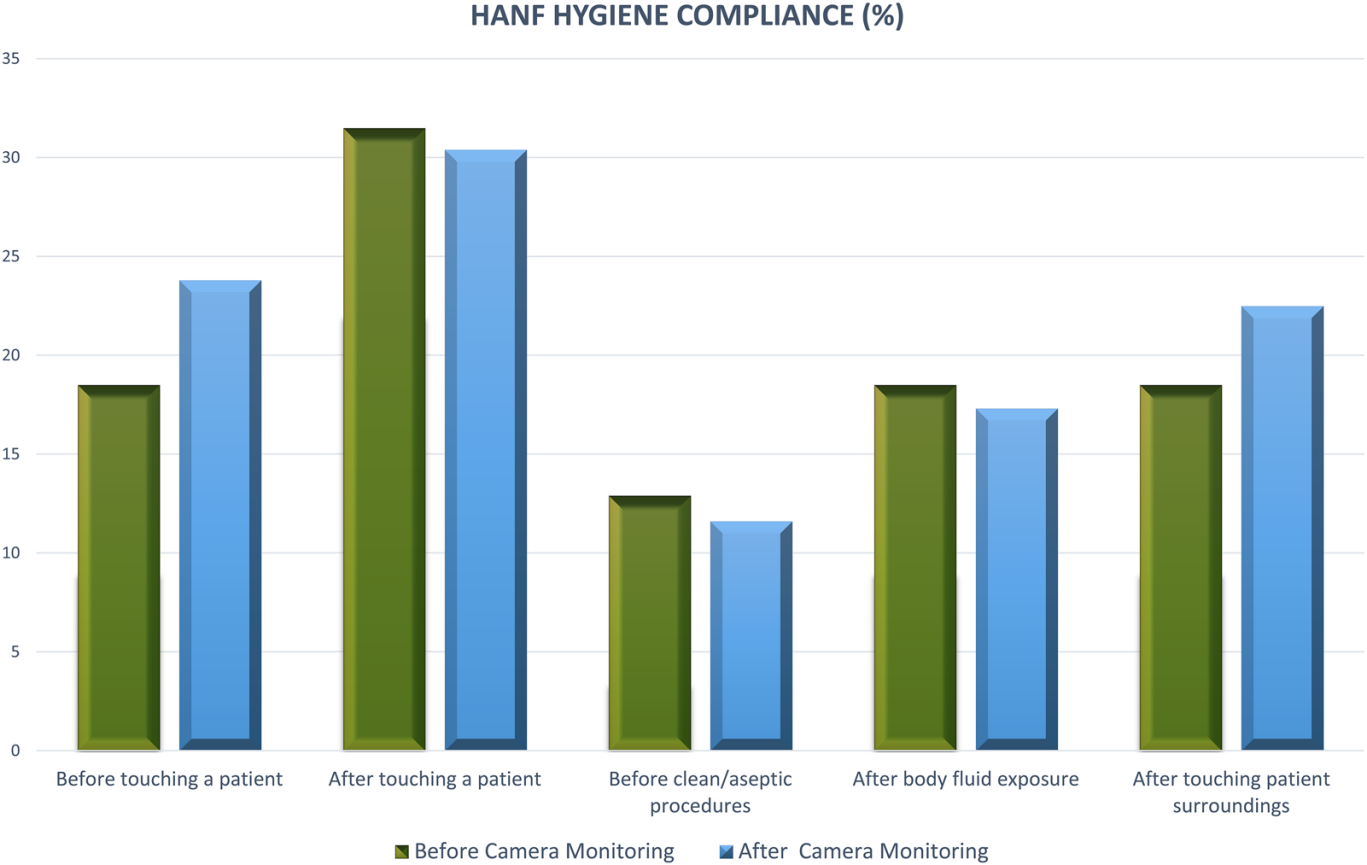
Hand hygiene compliance according to five indication rules before and after the camera observation

During the epidemic period, the rate of HAI was 9.59%, whereas this rate was decreased to 2.24% after the training and the CMAF. During the outbreak period, the HAI density was initially 5.01% and 1.26% after CMAF and statistically significant difference was observed between two time points (p < 0.01) (Table 1). Examining the HAI rates during epidemic and control periods, we pointed out that the rate of HAI and HAI density were reduced by 76.64% and 74.85%, respectively. In the same period, HCWs’ compliances with hand hygiene were evaluated. It was observed that the compliance rate was 49.0% (54/110) before the outbreak and 62.7% (69/110) after the training and CMAF (p = 0.041) (Fig. 1). According to the five indication rules, hand hygiene compliance data were given in Fig. 2. Compliance rates according to occupational status are presented in Table 2.

**Table 1:** Hospital infection rate and density before and after the camera observation

Parameters	Before camera monitoring	After camera monitoring	p value
Hospital infection rate (%)	9.59	2.24	< 0.01
Hospital infection density (‰)	5.01	1.26	< 0.01

**Table 2:** Hand hygiene compliance of health care workers

Occupations	Before camera monitoring	After camera monitoring	p value
Physicians (%)	50.00	68.18	0.220
Nurses (%)	58.33	70.83	0.200
Other healthcare personnel (%)	37.50	50.00	0.259

## Discussion

NICUs constitute great risk for neonatal infections. The infections in these units may easily be transmitted from one infant to another. A slightest deviation from the infection control in the NICU might yield serious consequences, which occasionally leads to infections and even outbreaks. We hypothesized that using the technology to monitor may reduce infection rate in the NICU. Hence, we used installed cameras to our NICU. In this study, a significant decrease in infection rate was observed in the CMAF period. Moreover, camera monitoring yielded a crucial increase in hand washing compliance. We demonstrated that HAI density was 9.59% before the onset of the CMAF, whereas it reduced to 2.24% during the CMAF period (p < 0.05). According to the results, CMAF process increased the compliance of infection control and improved hand hygiene habits in NICU. Previous studies declared that introducing CMAF to healthcare workers had great importance in hand hygiene [17]. Monitoring performed with the camera in intensive care units had been shown to improve hand washing rates significantly [17].

The concern of being watched made HCWs to work more meticulously. This is a crucial advantage to reduce hospital infections. In addition, reporting and providing feedbacks to units every week promoted employees to work more diligently. One of the most important results of this study was the 4 time decrease in the rate of infection during camera monitoring period. To the best of our knowledge, no such study has been conducted before in neonatal intensive care units. Similarly, in an observational study, Overdyk et al. [18] reported that video tracking and feedbacks have been shown to lead to a significant reduction in infection rates on a planned surgery. They reported that remote video auditing with feedback advance operating safety checklist compliance for all cases.

According to our observations, HCWs in this study were not pleased with the idea of being watched by a camera. Indeed, this is reasonable. Yet, the abovementioned dissatisfaction of CMAF decreased with time. Errors detected during the CMAF period were ameliorated by trainings. With feedbacks, HCWs were enabled to clarify the deficiencies in the unit, which became more convenient each day. Thus, prejudices were removed and infection control precautions evolved in a stricter aspect. Yet, it is also clear that it will be of concern as the monitoring was performed at work. It is obvious that working according to the rules will protect our patients towards infection risk.

In our study, general hand hygiene compliance in NICU was 49.1% and 62.7% before and after the CMAF period, respectively. In general, NICU comes from areas where hand hygiene is problematic. For instance, in our country, Karaaslan et al. [19] reported that the general hand hygiene compliance in NICU was only 37.0%. In our study, when hand hygiene compliance was evaluated according to occupations, after CMAF; that of doctors increased from 50.0 to 68.2%; that of nurses ascended from 58.3 to 70.8% and that of other health personnel elevated from 37.5 to 50.0%.

The presence of several factors facilitates the occurrence of infection in the neonatal unit. The main ones include the duration of intensive care, presence of invasive medical devices, parenteral nutrition, long term antimicrobial therapy, overcrowd and understaff, ward layout (sinks, bed spacing), use of contaminated scalp electrodes/probes, contact with colonized/infected family members, visitors, or healthcare workers, proximity to colonized neonates, increased length of stay [12]. To prevent infection in NICU; infection control measures must be adhered to without exception. Monitoring the employees is an effective way to improve compliance. However, employees should not feel pressured while doing so. Although technological developments carry out providing great range of options, the widely accepted hand hygiene compliance systems include direct observation, electronic or automated systems, and systems based on advanced technologies. Today still, direct observation is considered the gold standard in hand hygiene compliance. Despite ethical concerns, we also preferred observing the hand hygiene. However, in order to minimize ethical concerns, we informed all employees before the monitoring [20].

However, we must declare the limits of this study before making the final comment. This study includes a retrospective analysis of unplanned measures we have taken after an outbreak. Therefore, our recovery after the outbreak is the effect of the training, as well as the CMAF.

As a result, universal infection controls are essential in risky areas such as NICUs. However, neglecting these rules is a frequent behavior of the HCWs. In order to avoid this, continuing training is essential. We addressed that CMAF might be an important adjunct intervention in the prevention and control of NICU infections.

## Data Availability

No data or materials remained obscure. All information is clearly presented in the main manuscript. The datasets used and/or analyzed during the current study are available and can be provided by the corresponding author upon reasonable request.

## Abbreviations

NICU: neonatal intensive care units
HAIs: healthcare-associated infections
CDC: Center for Diseases Control
CMAF: camera monitoring and feedback
HCW: Health Care Personnel
